# Long noncoding RNA lncARSR confers resistance to Adriamycin and promotes osteosarcoma progression

**DOI:** 10.1038/s41419-020-2573-2

**Published:** 2020-05-13

**Authors:** Peng Shen, Yanfeng Cheng

**Affiliations:** Department of Dermatology, Shengjing Hospital of China Medical University, 36 Sanhao Street, Heping District, Shenyang, Liaoning 110004 China

**Keywords:** Bone cancer, Translational research

## Abstract

One of the significant challenges for chemotherapy is the appearance of resistance to compounds. Although several signaling pathways have been implicated in the development of Adriamycin (ADM) resistance, mechanisms involved in ADM-resistant osteosarcoma progression remain unknown. The present study attempted to illustrate the role of long noncoding RNA ARSR (lncARSR) in the development of adapted ADM resistance. We found lncARSR overexpressed in the Adriamycin-resistant cell lines U2OS/ADM and MG63/ADM, accompanied with acquired multidrug resistance against to paclitaxel and cisplatin. Overexpression of lncARSR triggered rhodamine 123 efflux and survival, as well as the migration of Adriamycin-resistant cells. Inversely, the depletion of lncARSR promoted rhodamine 123 retention and apoptosis, while reducing the motility of ADM-resistant cells. Further investigation revealed that the upregulation of lncARSR enhanced multidrug resistance-associated protein-1 (MRP1), apoptosis inhibitor Survivin, and matrix metalloproteinase-2 (MMP2) through activating AKT. The reduction of lncARSR overcame the resistance to ADM in U2OS/ADM mouse model. The current study gained novel evidence for understanding the mechanisms underlying adaptive ADM resistance and provided rationales to improve clinical outcomes of refractory osteosarcoma.

## Introduction

Since the MAP (methotrexate, Adriamycin, and cisplatin) chemotherapy was introduced into the treatment for patients suffered from osteosarcoma, it is unchanged over the past decades^1^. Clinical outcomes, remaining 65–70%, have similarly improved little over the last decades^2^. According to the development of immunotherapy and targeted therapy, patients received combination therapy, including surgical resection, chemotherapy, radiotherapy, and targeted therapy, are expected to prolong survival. Nevertheless, chemoresistance obstructs the response of relapse or metastasis of osteosarcoma to chemotherapy. There is an imperative need for verifying the details of chemoresistance.

Long noncoding RNAs (lncRNAs) are RNA molecules with more than 200 nucleotides while lacking protein-coding capacity. Abnormal expression of lncRNAs is involved in dysregulation of different biological functions in cancer initiation, progression, and recurrence, such as proliferation, migration and invasion, stemness, radio-resistance, and chemoresistance^3^. The mechanisms underlying different lncRNAs were discrepant. Previous studies have demonstrated that lncRNA FENDRR, LINC00161, and CTA enhanced sensitivity to active chemical agents^4–6^, whereas the others induced resistance via multiple signaling pathways.

LncRNAs play dual roles in regulating gene expression by various mechanisms. As oncogenes, the expression of H91antisense RNA (91H) elevated in osteosarcoma and correlated with osteosarcoma progression. Impairment of 91H reduced proliferation and promoted apoptosis^7^. MALAT-1, metastasis-associated lung adenocarcinoma transcript 1, enhanced PI3K/AKT activation and led to osteosarcoma metastasis^8^. On the other hand, lncRNAs work as tumor suppressors. For instance, the negative-feedback loop of Loc285194 and miR-211 played an important role in osteosarcoma development^9^.

Recently, lncRNA Activated in RCC (renal cell carcinoma) with Sunitinib Resistance, lncARSR, was found to promote resistance against sunitinib in renal cancer^10^ and triggered tumor cell dissemination^11^. Furthermore, lncARSR enhanced hepatocellular carcinoma resistance to Adriamycin (ADM) and steatohepatitis via activation of AKT^12,13^. Besides, overexpression of lncARSR maintained cancer stem cells in liver cancer by activating STAT3-mediated pathways^14^. Nevertheless, the knowledge of lncARSR in osteosarcoma is little. The present study found lncARSR expression raised in ADM-resistant osteosarcoma cells and aimed to illuminate the mechanisms underlying lncARSR increment and ADM resistance of osteosarcoma.

## Materials and methods

### Chemicals and reagents

Adriamycin (Doxorubicin, ADM), paclitaxel (PTX), and cisplatin (DDP) were obtained from Selleck Chemicals (Houston, TX, USA). Lipofectamine RNAiMAX and dimethyl sulfoxide (DMSO) were purchased from Thermo Fisher Scientific (Waltham, MA, USA). siRNA negative control and siRNA targeting lncARSR were synthesized by OriGene. shRNA negative control and shRNA targeting lncARSR were inserted into pGFP-C-shLenti vector and amplified according to the manufacturer’s instructions (OriGene, Rockville, MD, USA).

### Cell culture

Human osteosarcoma cell lines U2OS and MG63 were purchased from Cell Bank of the Chinese Academy of Sciences (Shanghai, China). Cells were cultured in RPMI-1640 supplemented with 10% fetal bovine serum (Thermo Fisher Scientific, Waltham, MA, USA) and were passaged upon reaching 70–80% confluency.

### Generation of Adriamycin-resistant cells

U2OS and MG63 cells were exposed to intermittent and continue the stepwise increase of ADM concentrations from 0.2 mg/l ADM. Twenty-four hours later, ADM was removed, and cells were cultured with fresh medium until the proliferation rate returned to the identical rate of parental cells. At this point, the ADM concentrations increased and repeated the above process. During this 6-month-long period, the ADM-resistant subline of each cell line named U2OS/ADM and MG63/ADM were induced, respectively.

### LncRNA microarray analysis

Total RNA from parental or resistant cells was used for RT2 LncRNA Array (QIAGEN N.V. Hilden, German). LncRNA microarray analysis was performed according to the manufacturer’s protocol. LncRNAs (fold change >1.5, and *P*-value <0.05) were considered expressed distinctively between two groups. Three independent lncRNA arrays were performed. Data obtained from the lncRNA microarray was curated and approved by Gene Expression Omnibus (Accession no. GSE142230).

### Rhodamine 123 accumulation assay

P-glycoprotein (P-gp) inhibitory potential was measured by the accumulation of rhodamine 123 in cells in the absence or presence of ADM. Details were described previously^15^. To measure the rhodamine 123 (Rh123) inhibitory potential of the ADM-resistant cells and their corresponding parental cells, 5 × 10^5^ cells were seeded in 6-well plates. Twelve hours later, rhodamine 123 accumulation assay was performed following flow cytometry analysis. Data presented as a fold change of rhodamine 123-associated median fluorescence intensity (MFI).

### MTT assay

Cell viability was determined by MTT assay, as previously mentioned^16^. Cells were plated with 0.1 ml RPMI-1640 supplemented 10% fetal bovine serum, together with DMSO or ADM. Ten microliters of MTT solution (5 mg/ml) was complemented and followed by 4-h-long incubation before measurement. A Microplate Reader (Life Science, Hercules, CA, USA) was used to collect the optical densities at 490 nm (OD490nm). Data gained from three independent experiments. IC_50_ values were estimated as the drug concentration required for inhibiting 50% cell growth. Resistance index (RI) = IC_50_ of resistant cells/IC_50_ of parental cells.

### JC-1 apoptosis analysis

Cell apoptosis was analyzed by a JC-1 assay kit (Beyotime, Shanghai, China) followed by flow cytometry (Beckham Coulter, Brea, CA, USA). Cells were plated in 6-well plates and allowed to attach overnight. JC-1 assay was conducted post-ARSR transfection 24 h according to the manufacturer’s protocols. Results from three independent experiments were analyzed with Kaluza Analysis Software 2.1.1 (Beckham Coulter, Brea, CA, USA).

### Migration assay

Transwell assay was performed using modified Boyden chambers without matrigel (Merck & Co., Inc., Kenilworth, NJ, USA) to measure the cell migration. Cells in 0.2 ml serum-free RPMI-1640 were seeded in the upper room of each chamber, while the lower room was filled with 0.6 ml DMEM supplemented with 10% FBS. After incubating for 24 h at 37 °C, cells on the upper compartments were removed, whereas the migrated cells in the lower parts were stained with 0.4% (w/v) Trypan blue solution, observed and counted under a ×100 magnification microscope.

### Immunoblots

Total proteins were extracted using 100 μl lysis buffer form cells. After measurement of the quality and concentrations of the proteins, 25 μg samples were loaded and separated in 10% sodium lauryl sulfate-polyacrylamide gel electrophoresis (SDS-PAGE). Proteins were transferred to nitrocellulose membranes through electroblotting. Then membranes were blocked with 5% skim milk for 1 h at room temperature, followed by incubation with P-gp (Cat. No. MA5-13854, Invitrogen, Carlsbad, CA, USA), MRP1 (SC-18835), Survivin (sc-17779), MMP2 (sc-13594), pSer473-Akt (sc-293125), Akt1 (sc-5298), p-ERK (sc-7383), ERK (sc-514302), pSer33/37-β-catenin (sc-57535), β-catenin (sc-7963), p-NFκB (sc-271908), NFκB (sc-8414), p-mTOR (sc-293089), mTOR (sc-517464), and GAPDH (SC-47724) antibodies (Santa Cruz Biotechnology, Inc., Dallas, TX, USA) overnight at 4 °C in a dilution of 1:1000. Membranes were washed three times with phosphate-buffered saline Tween-20 and incubated with HRP-conjugated secondary antibody (Merck & Co., Inc., Kenilworth, NJ, USA) for another 1 h in a dilution of 1:2500. Immunoreactivity was detected using the Western Lighting Ultra (Thermo Fisher Scientific, Waltham, MA, USA).

### Quantitative real-time polymerase chain reaction (qRT-PCR)

Total RNA was isolated with an RNA Isolation Kit (QIAGEN N.V. Hilden, German) according to the manufacturer’s protocol. One milligram RNA was reverse transcribed to cDNA by QuantiTect Reverse Transcription Kit (QIAGEN N.V. Hilden, German). Real-time PCR was carried out with the Mx3000P real-time PCR system (Thermo Fisher Scientific, Waltham, MA, USA). The protocols were as follows: 40 cycles of 94 °C for 15 s, 60 °C for 10 s, and 72 °C for 20 s. All procedures repeated thrice. Gene expression was normalized to the GAPDH to calculate relative expression using the 2^−ΔΔCq^ method^17^. Primers for detecting *lncARSR*, *MRP1, SURVIVIN*, *MMP2 AKT*, and *mTOR*, listed below:

LncARSR, forward: 5ʹ-TTTGAAATGCTCTTTGAGGGAT-3ʹ; reverse: 5ʹ-TGCAGGTTGTCTGAAGTTGGA-3ʹ. *MRP1*, forward: 5ʹ-ACCCTAATCCCTGCCCAGAG-3ʹ, reverse: 5ʹ-CGCATTCCTTCTTCCAGTTC-3ʹ. *SURVIVIN*, forward: 5′-AAGAACTGGCCCTTCTTGGA-3ʹ, reverse: 5′-CAACCGGACGAATGCTTTT. *MMP2*, forward: 5ʹ-AGCGAGTGGATGCCGCCTTTAA-3ʹ, reverse: 5ʹ-CATTCCAGGCATCTGCGATGAG-3ʹ. *AKT*, forward: 5ʹ-TGGACTACCTGCACTCGGAGAA-3ʹ, reverse: 5ʹ-GTGCCGCAAAAGGTCTTCATGG-3ʹ. *MTOR*, forward: 5ʹ-AGCATCGGATGCTTAGGAGTGG-3ʹ, reverse: 5ʹ-CAGCCAGTCATCTTTGGAGACC-3ʹ. *GAPDH*, forward: 5ʹ-GTCTCCTCTGACTTCAACAGCG-3ʹ, reverse: 5ʹ-ACCACCCTGTTGCTGTAGCCAA-3ʹ.

### Generation of U2OS/ADM human osteosarcoma mouse models

All studies were approved by the medical ethical committee of Shengjing Hospital of China Medical University and conducted according to the guidelines of the Centre of Experimental animal of Shengjing Hospital of China Medical University. Four-week-old female BALB/c nude mice were purchased from Nanjing Biomedical Research Institute of Nanjing University. 3 × 10^6^ U2OS/ADM cells expressing shRNA negative control or shRNA targeting lncARSR in 200 µl saline were injected into nude mice subcutaneously. Seven days later, 24 mice with ~100-mm^3^ tumors were grouped randomly (six per group) and received 6 mg/kg ADM or saline by intraperitoneal injection once weekly. The body weights of the mice, as well as the tumor volumes, were measured every 4 days. The tumor volume was estimated using the formula *V* = 1/2 (width^2^ × length). The in vivo experiments terminated at day 28 post-treatment with ADM and mice were euthanasia. Tumors were resected and weighed. Immunohistochemical staining was performed followed the standard procedure^18^.

### Statistical analysis

The data from all experiments were shown as means plus standard deviation. The differences were evaluated by one-way analysis of variance (ANOVA) with LSD test, and *P* < 0.05 was statistically significant. Statistical analysis was conducted using GraphPad version 7.0 (San Diego, CA, USA).

## Results

### Establishment of Adriamycin-resistant osteosarcoma cells

**Fig. 1:**
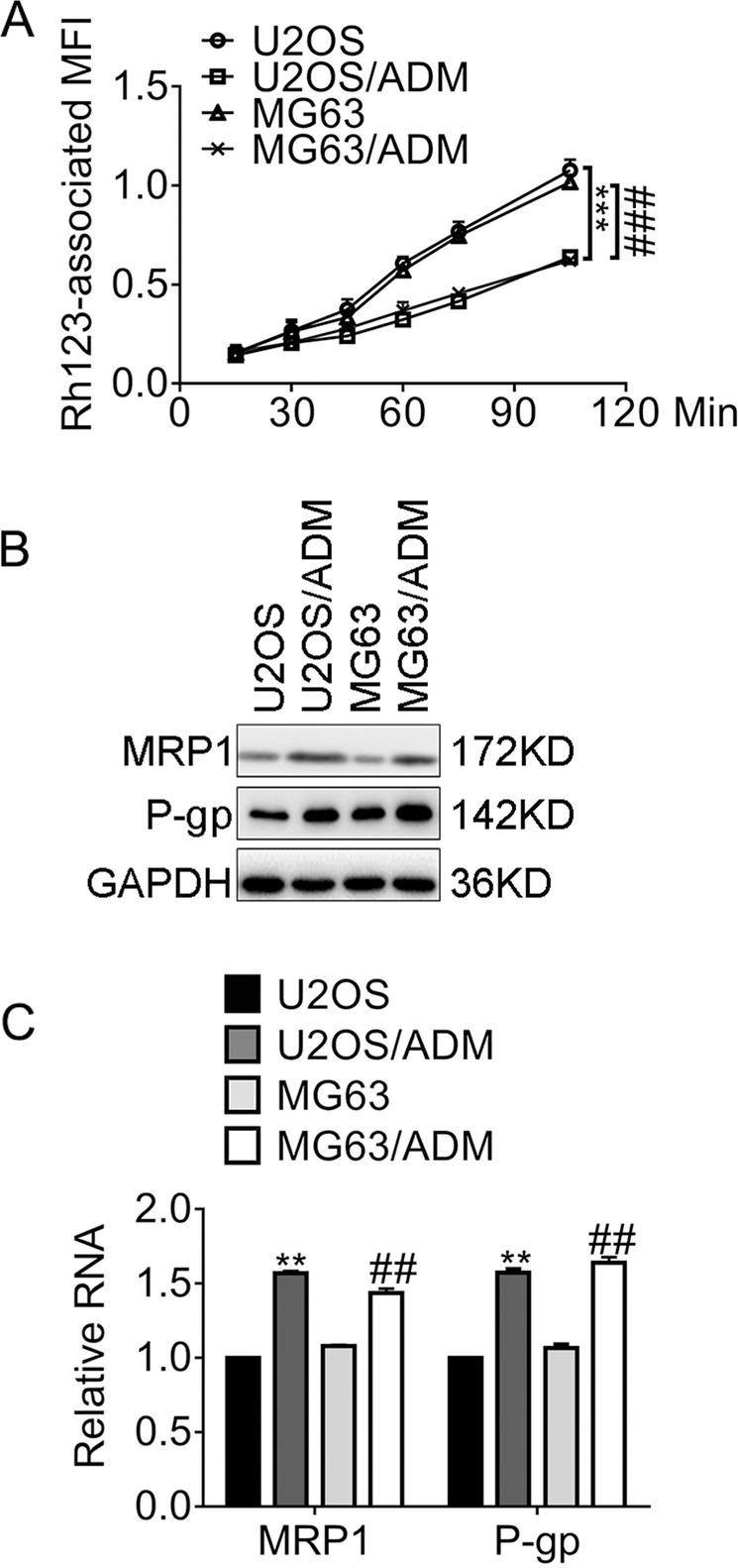
Resistance profiles of Adriamycin-resistant cells. **a** The rhodamine 123-associated median fluorescence intensity (MFI) of the indicated cells. ***P < 0.001, vs. U2OS; ^###^*P* < 0.001, vs. MG63. **b** The expression of the indicated proteins was accessed by immunoblots. **c** The expression of the indicated genes was accessed by qRT-PCR analysis. **P < 0.01, vs. U2OS; ^##^*P* < 0.01, vs. MG63. Data obtained from at least three independent experiments and presented as means plus standard deviation.

To evaluate the resistance to ADM, we calculated the resistance index (RI) by estimating the ratio of the half-maximal inhibitory concentration (IC_50_) of ADM-resistant cells over parental cells. Table 1 exhibited the IC_50_ and the corresponding RI values of each cell line. The RI values of U2OS/ADM and MG63/ADM increased by 95- and 72-fold, respectively. Because of the frequent cross-resistance to different agents of chemo-resistant osteosarcoma cells, we further conducted MTT assay with ADM-resistant cells exposed to paclitaxel (PTX) and cisplatin (DDP). Table 2 showed the IC_50_ and the corresponding RI values of each cell line against PTX and DDP. The RI values of U2OS/ADM against PTX and DDP were 3.01- and 3.06-fold stronger than the parental cell line at the ID_50_ level, separately. Similarly, the RI values of MG63/ADM against PTX and DDP were 2.95 and 3.03, separately. The above results revealed that the ADM-resistant cells gained multidrug resistance (MDR). To determine the P-glycoprotein (P-gp) inhibitory potential, we selected the parental osteosarcoma cells and the ADM-resistant cells to perform the rhodamine 123 accumulation assay. Figure 1a exhibited the rhodamine 123-associated median fluorescence intensity (MFI) of U2OS/ADM cells decreased significantly compared to U2OS cells (0.64 vs. 1.08, *P* < 0.001). Likewise, the Rh123-associated MFI of MG63/ADM reduced markedly in comparison with MG63 cells (0.62 vs. 1.62, *P* < 0.001). According to the identical efflux curves, the activity of P-gp increased in ADM-resistant cells compared with that in the parental cells. Therefore, we explored the multidrug resistance-associated protein-1 (MRP1) and the apoptosis inhibitor Survivin in the parental- and ADM-resistant cells. Figure 1b and c showed that MRP1 and P-gp was upregulated in U2OS/ADM at protein level and mRNA level, separately. Similar results were observed in MG63/ADM cells compared to MG63 cells. In brief, the results displayed the resistance profiles of U2OS/ADM and MG63/ADM cells.

**Table 1:** IC_50_ and RI values of the indicated cell lines against Adriamycin.

Cell line	IC_50_	RI
U2OS	0.14 ± 0.03	1
U2OS/ADM	13.38 ± 0.54	95.57
MG63	0.18 ± 0.02	1
MG63/ADM	13.12 ± 1.11	72.89

Unit: µmol/l.

*IC*_*50*_ the half-effective inhibition concentrations, *RI* resistance index.

**Table 2:** IC_50_ and RI values of the indicated cell lines against PTX or DDP.

	IC50				RI	
	U2OS	U2OS/ADM	MG63	MG63/ADM	U2OS/ADM	MG63/ADM
PTX	3.48 ± 0.12	10.46 ± 0.04	4.11 ± 0.24	12.14 ± 0.15	3.01	2.95
DDP	4.07 ± 0.81	12.47 ± 0.72	5.07 ± 0.32	15.37 ± 0.42	3.06	3.03

Unit: µmol/l.

*IC*_*50*_ the half-effective inhibition concentrations, *RI* resistance index. Unit, µmol/l.

*PTX* paclitaxel, *DDP* cisplatin.

### LncRNA ARSR increases in ADM-resistant osteosarcoma cells and enhances cell survival exposed to ADM

**Fig. 2:**
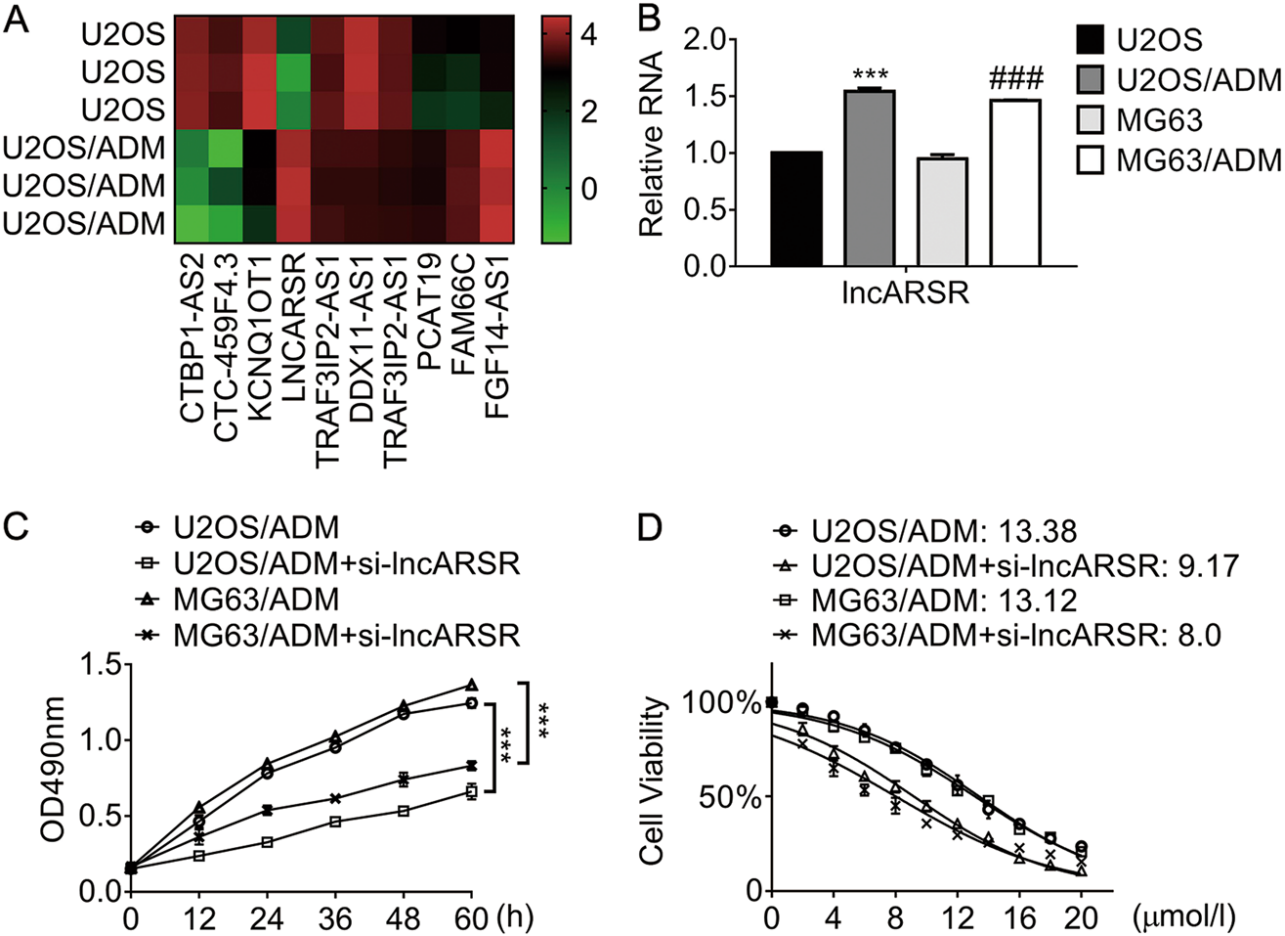
LncARSR increases in Adriamycin-resistant cells and relates to resistance. **a** The heatmap showed the distinct expression of lncRNA in U2OS and U2OS/ADM cells. **b** The expression of lncARSR in ADM-resistant cells and parental cells was examined by qRT-PCR. ***P < 0.001, vs. U2OS; ^###^*P* < 0.001, vs. MG63. **c** The viability of the indicated cells was detected by MTT assay. Cells were transfected with si-lncARSR or siRNA negative control, and MTT assay was conducted at different time-points. ***P < 0.001, vs. U2OS/ADM or MG63/ADM cells, respectively. **d** The viability of the indicated cells post-transfection of si-lncARSR or siRNA negative control was accessed by MTT assay. The half-effective inhibition concentrations (IC_50_) were calculated according to the data obtained from three independent MTT assays. Data obtained from at least three independent experiments and presented as means plus standard deviation.

In the light of the analysis of lncRNA microarray data, we found several distinctly expressed lncRNA in U2OS/ADM cells (Fig. 2a), including lncARSR. Consistent with the results from microarray, the expression of lncARSR raised apparently in ADM-resistant cells compared with the corresponding parental cells (Fig. 2b). Moreover, knockdown of lncARSR in U2OS/ADM cells suppressed cell growth evidently compared to that expressing scrambled siRNA (Fig. 2c) in a time-dependent manner. In addition, the downregulation of lncARSR in the resistant cells reduced the IC_50_ values against ADM. As the results shown in Fig. 2d, the IC_50_ values of U2OS/ADM expressing si-lncARSR decreased by 30% approximately, and the IC_50_ values of MG63/ADM expressing si-lncARSR dropped by 40% compared with their parental cells. The results in Fig. 2 indicated that lncARSR was essential for the resistance to ADM.

### Overexpression of lncARSR promotes the viability and migration while antagonizes apoptosis of osteosarcoma cells

**Fig. 3:**
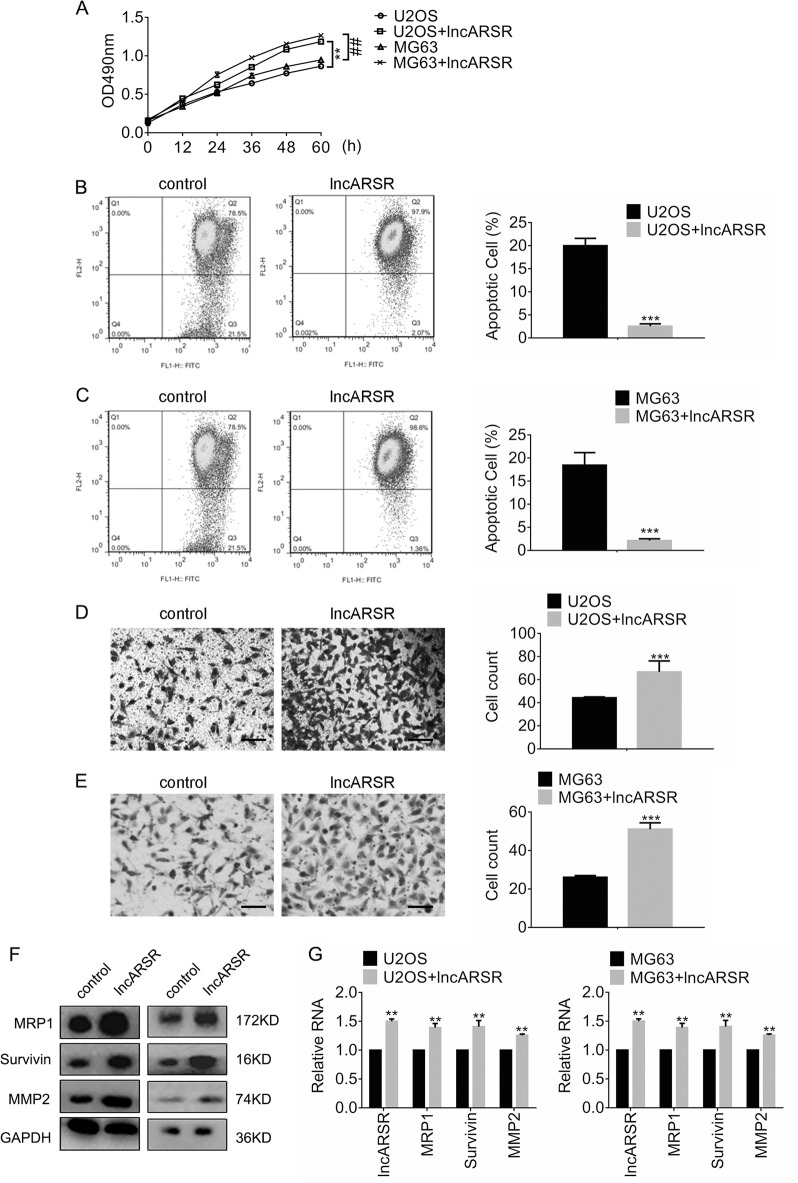
Overexpression of lncARSR promotes the viability and migration of the parental osteosarcoma cells, while antagonizes apoptosis. **a** The viability of cells expressing control or LncRNA ARSR was determined by MTT assay. **P < 0.01, vs. U2OS plus control; ^##^*P* < 0.01, vs. MG63 plus control. Apoptosis of (**b**) U2OS and (**c**) MG63 cells expressing control or LncARSR was accessed by JC-1 assay following by flow cytometry analysis. ***P < 0.001, vs. control. Migration of (**d**) U2OS and (**e**) MG63 cells were detected by transwell assay. ***P < 0.001, vs. control. Scale bar, 200 μm. Magnification, ×100. **f** The indicated protein expression in U2OS and MG63 cells were analyzed by immunoblots. **g** The expression of the indicated lncRNA and mRNA in U2OS and MG63 cells were analyzed by qRT-PCR. **P < 0.01, vs. control. Data obtained from at least three independent experiments and presented as means plus standard deviation.

To investigate the role of lncARSR in resistance against ADM, we introduced lncARSR into U2OS and MG63 cells. The viability of cells expressing lncARSR was accessed by MTT assay afterward. Figure 3a showed that overexpression of lncARSR promoted viability of U2OS and MG63 cells compared to control. Furthermore, the percentage of apoptotic U2OS cells decreased from 20.01 to 2.05% in the presence of ADM (Fig. 3b). Consistently, the results in Fig. 3c showed the apoptotic MG63 cells expressing lncARSR fell significantly post-exposure to ADM (control, 17.08% vs. lncARSR, 1.93%). In addition, Fig. 3d demonstrated that the median counts of migrant U2OS cells expressing lncARSR increased apparently (lncARSR, 66 vs. control, 44). Similarly, Fig. 3e exhibited the number of migrated MG63 cells raised from 26 (control) to 51 (lncARSR). To explore the mechanisms underlying the alterations, we determined the expression of MRP1, Survivin and matrix metalloproteinase-2 (MMP2) by immunoblots and qRT-PCR, separately. The results in Figs. 3f and g showed the ectopic expression of lncARSR triggered expression of MRP1, Survivin, and MMP2 both at protein- and mRNA levels.

### Downregulation of lncARSR hampers the viability and motility while inducing rhodamine 123 accumulation and apoptosis of ADM-resistant cells

**Fig. 4:**
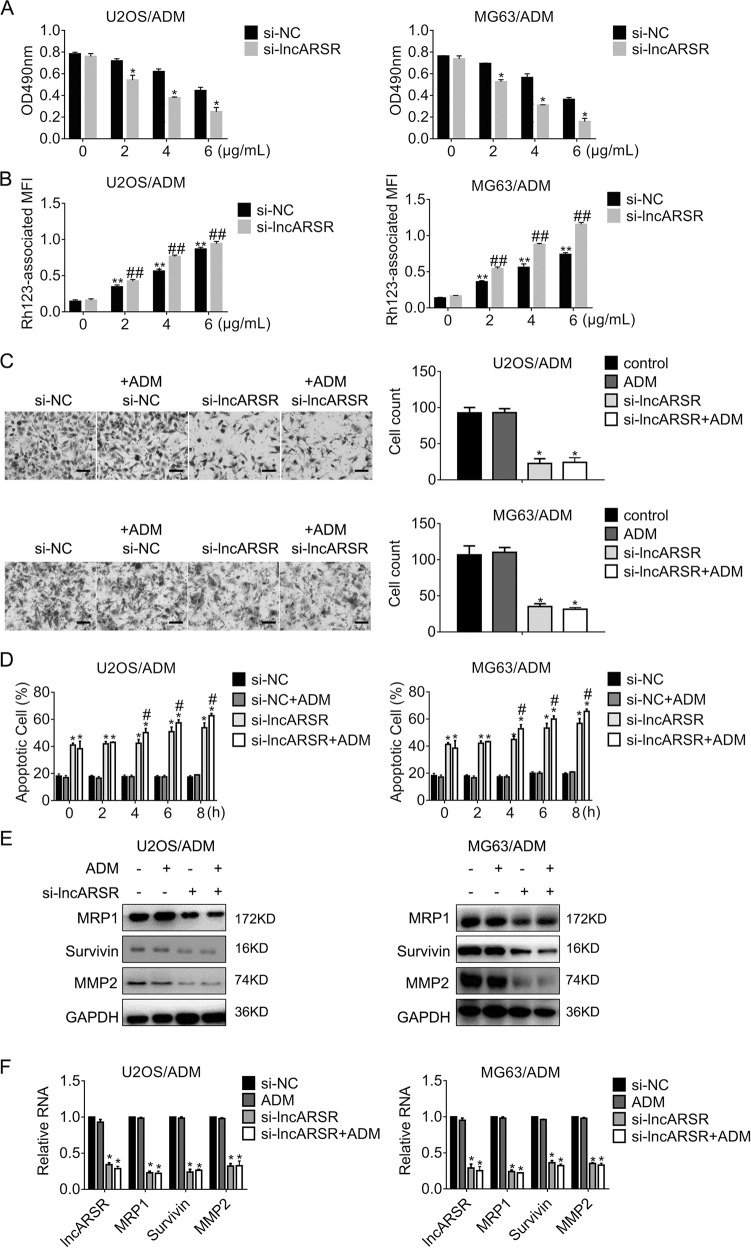
Reduction of lncARSR impairs the growth, rhodamine 123 efflux, and migration of the Adriamycin-resistant cells while enhancing apoptosis. **a** Cell viability of U2OS/ADM or MG63/ADM cells expressing scramble siRNA or si-lncARSR was detected by MTT assay. *P < 0.05, vs. si-NC. **b** Rh123 retention of U2OS/ADM and MG63/ADM cells expressing si-NC or si-lncARSR was accessed by rhodamine 123 assay and flow cytometry analysis. **P < 0.01, vs. si-NC without ADM. ^##^*P* < 0.01, vs. si-NC plus ADM. **c** The migration of U2OS/ADM cells were detected by transwell assay, respectively. *P < 0.05, vs. si-NC. Scale bar, 200 μm. Magnification, ×100. **d** The apoptosis of U2OS/ADM or MG63/ADM cells by JC-1 assay followed by flow cytometry analysis. Cells were transfected with siRNA targeting lncARSR. Twenty-four hours later, cells were exposed to 2 µg/ml ADM, and apoptosis was measured at different time-points. *P < 0.05, vs. si-NC; ^#^*P* < 0.05, vs. si-NC plus ADM. **e** The indicated genes expression in U2OS/ADM and MG63/ADM cells in the presence or absence of ADM were detected by western blot, separately. **f** The indicated genes expression in MG63/ADM cells in the presence or absence of ADM were detected by western blot and real-time PCR, separately. si-NC, siRNA negative control. Data obtained from at least three independent experiments and presented as means plus standard deviation.

Apart from gain-of-function experiments, we carried out loss-of-function experiments to evaluate the effects of lncARSR on ADM resistance. The viability of resistant cells lacking lncARSR was determined by MTT assay. As shown in Fig. 4a, the optical density at 490 nm fell apparently in cells expressing siRNA targeting lncARSR cells while that expressing siRNA negative control remained stable in an ADM doses-dependent manner. Similar results were gained in MG63/ADM cells. Furthermore, lncARSR-knockdown cells were exposed to increasing concentrations of ADM, and the Rh123 accumulation was accessed. The results in Fig. 4b indicated that si-lncARSR promoted Rh123 retention in an ADM dose-dependent manner. To observe the effects of lncARSR on cell motility, we conducted transwell assay without matrigel. Figure 4c proved that attenuation of lncARSR repressed cell migration significantly. In addition, lncARSR knockdown enhanced ADM-triggered apoptosis in a time-dependent manner (Fig. 4d). To examine the details of lncARSR-induced alterations, we measured the expression of MRP1, Survivin, and MMP2 was detected by immunoblots and qRT-PCR, respectively. As the results shown in Fig. 4e, the diminishment of lncARSR repressed the expression of MRP1, Survivin, and MMP2. In line with the changes of protein, the expression of lncARSR and the interesting mRNA declined significantly in cells expressing si-lncARSR (Fig. 4f).

### Hindrance of lncARSR expression obstructs tumor growth and restores ADM-induced growth inhibition in U2OS/ADM human osteosarcoma mouse models

**Fig. 5:**
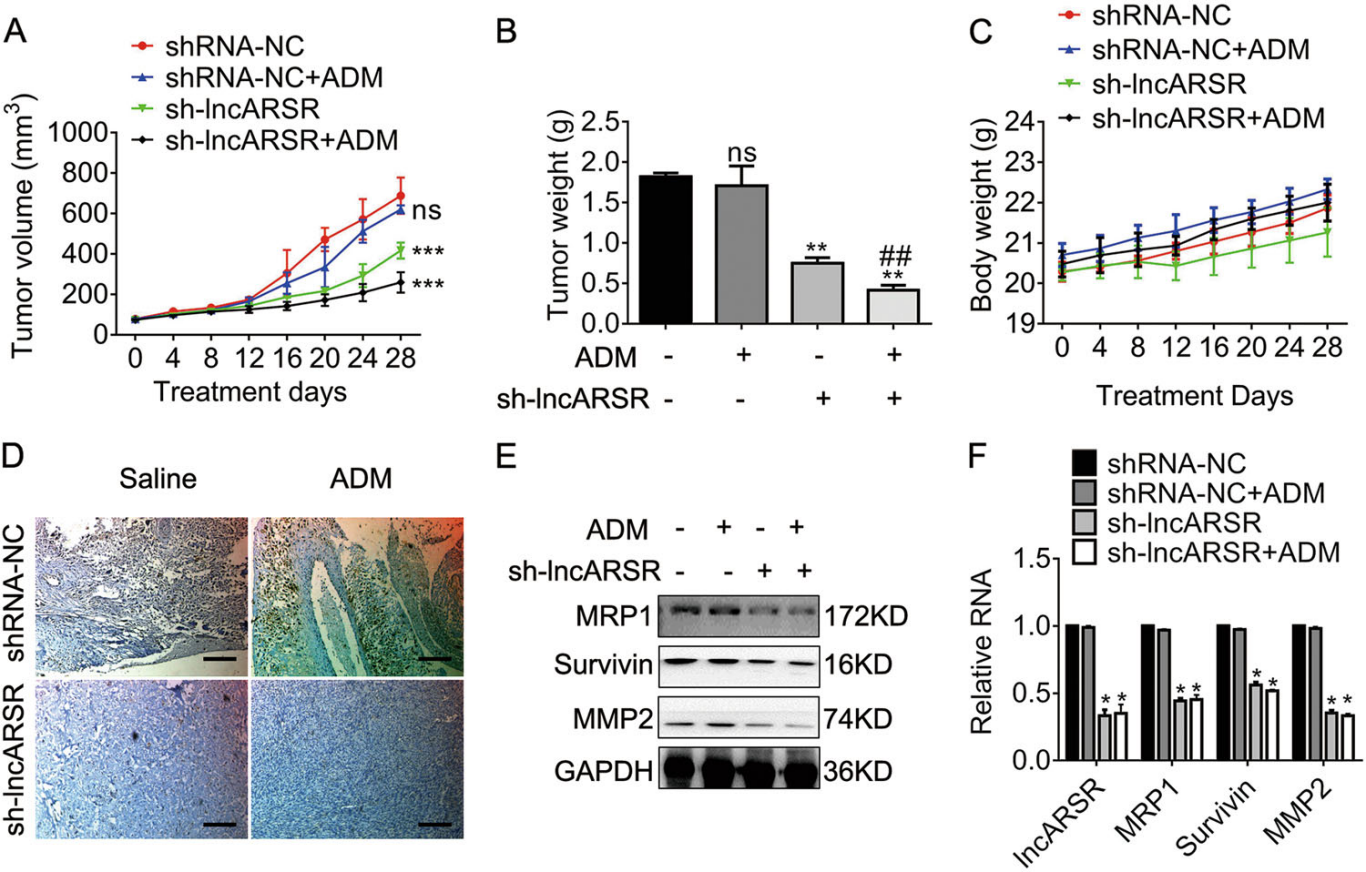
The knockdown of lncARSR suppresses tumor growth via promoting sensitivity to ADM. **a** Tumor volumes of U2OS/ADM mouse models. Mouse in each group received 6 mg/kg ADM or equal volumes of saline by intraperitoneal injection once per week. ***P < 0.001, vs. shRNA-NC. ns, no significance, vs. shRNA-NC. **b** Tumor weights of U2OS/ADM mouse models 28 days after treatment with ADM. **P < 0.01, vs. shRNA-NC. ns, no significance, vs. shRNA-NC. ^##^*P* < 0.01, vs. sh-lncARSR. **c** Body weights of U2OS/ADM mouse models. **d** Representative images for immunohistochemical staining of MRP1. Scale bar, 200 μm. Magnification, ×100. **e** The indicated protein expression in tumors was accessed by immunoblot. **f** The expression of the indicated lncRNA and mRNA in tumors was measured by qRT-PCR. *P < 0.05, vs. shRNA-NC. shRNA-NC, shRNA negative control. Data obtained from at least three independent experiments and presented as means plus standard deviation.

To evaluate the potential effects of si-lncARSR plus ADM on tumor growth, we subcutaneously injected U2OS/ADM cells to BALB/c nude mice to generate ADM-resistant osteosarcoma mouse models. Mice harbored approximately 100-mm^3^ tumors received 6 mg/kg ADM or equal saline by intraperitoneal injection once a week according to the previous protocols^19^. The tumor volumes were measured every 4 days, as well as the body weights. Figure 5a demonstrated that the deregulation of lncARSR suppressed tumor growth significantly compared to siRNA negative control. Importantly, si-lncARSR recovered the suppression of tumor growth by ADM. The alterations of tumor weights were consistent with those of tumor volumes (Fig. 5b). The increment of body weights among divergent groups showed no significant difference (Fig. 5c), suggested that the doses of the combination regimens were tolerant. The MRP1 expression in tumors was accessed by immunohistochemistry further. Figure 5d illuminated the MRP1 expression reduced notably in tumors deregulated lncARSR compared to that in tumors with siRNA negative control. Consistent with the previous in vitro experiments, the expression of MRP1, Survivin, and MMP2 fell remarkably in tumors without lncARSR (Fig. 5e and f).

### Interference of lncARSR reverses resistance to ADM and represses tumor malignancy via hindering AKT activation

**Fig. 6:**
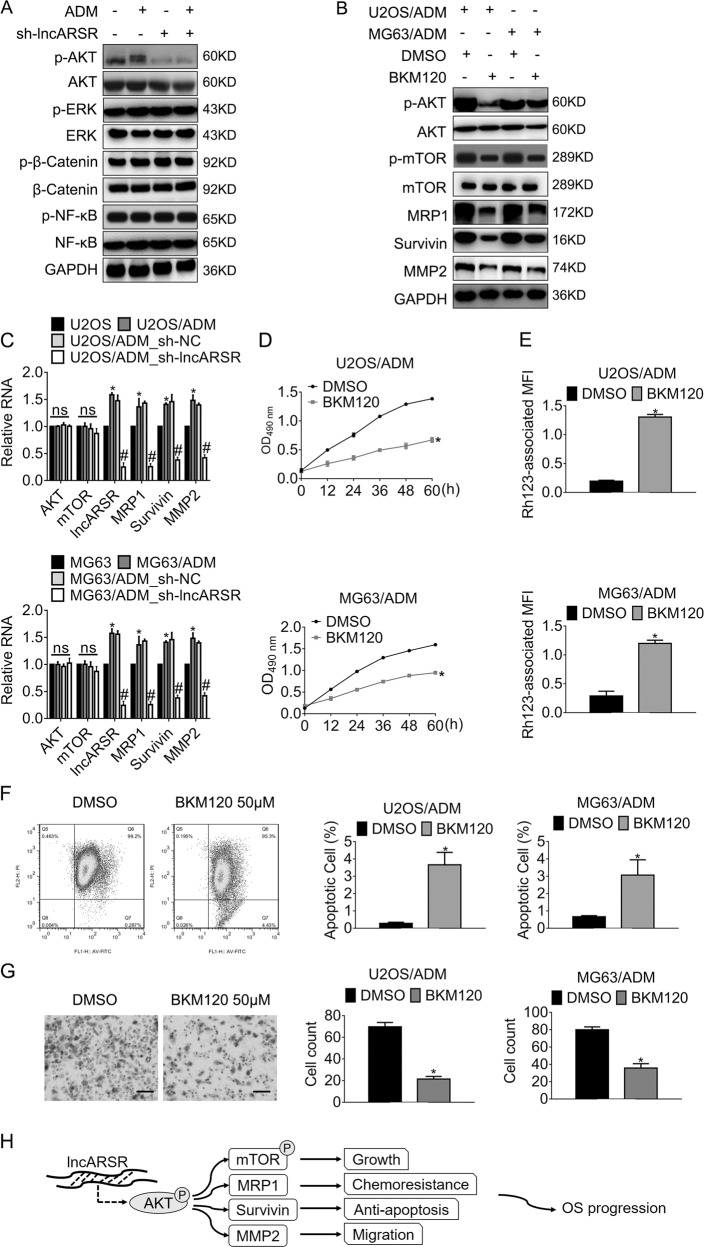
Interference of lncARSR impedes resistance against ADM and retards tumor progression in an Akt-dependent manner. **a** The expression of the indicated protein in U2OS/ADM was detected by western blot. **b** The expression of the indicated protein in ADM-resistant cells was detected by western blot. **c** The expression of the indicated genes was examined by qRT-PCR. *P < 0.05, vs. parental cells. ^#^*P* < 0.05, vs. ADM-resistant cells with sh-NC. **d** The growth of the indicated cells was accessed by MTT assay. *P < 0.05, vs. DMSO. **e** Rh123 retention of the indicated cells was accessed by rhodamine 123 assay, followed by flow cytometry analysis. *P < 0.05, vs. DMSO. **f** The apoptosis of the indicated cells was analyzed by JC-1 assay and flow cytometry analysis. *P < 0.05, vs. DMSO. **g** The migration of the indicated cells was accessed by transwell assay. *P < 0.05, vs. DMSO. Scale bar, 200 μm. Magnification, ×100. **h** The schematic pathways of lncARSR confers ADM resistance and promotes OS malignancy via activating AKT-mediated cascades. The aberrant expression of lncARSR activates AKT, subsequently enhances mTOR phosphorylation and the expression of MRP1, Survivin, and MMP2, leading to cell growth, acquisition of chemoresistance, survival, and migration. ADM, Adriamycin. OS, osteosarcoma. Cells were exposed to pan-PI3K inhibitor BKM120 50 μM for 24 h before analysis. Data obtained from at least three independent experiments and presented as means plus standard deviation.

We investigated the phosphorylation of the frequently hyperactivated kinases in osteosarcoma, including PI3K/AKT^20^, MAPK/ERK^21^, β-Catenin^22^, and NFκB^23^. The results in Fig. 6a displayed that the phosphorylation of AKT in U2OS/ADM xenograft models dropped, while the expression of total AKT changed little. Meanwhile, the activation of ERK, β-Catenin, and NFκB changed hardly. The pan-PI3K inhibitor BKM120 was subsequently introduced into the ADM-resistant sublines. Figure 6b showed that the inhibition of AKT caused dephosphorylation of mTOR (Mammalian Target of Rapamycin), which was the direct target of AKT and played a central role in the regulation of cellular growth and survival. Moreover, the expression of MRP1, Survivin, and MMP2 reduced, accompanied by the deregulation of AKT. In line with expectations, the alterations of AKT and mTOR at mRNA level were consistent with that at protein level (Fig. 6c). The exposure of BKM120 suppressed cellular growth (Fig. 6d) and rhodamine 123 cellular retention (Fig. 6e), whereas promoting apoptosis (Fig. 6f). The migration of ADM-resistant sublines decreased post-treatment of BKM120 as well (Fig. 6g). The mechanisms that lncARSR conferred ADM resistance and promoted osteosarcoma progression via activating AKT were summarized in Fig. 6h.

## Discussion

The most common cancer of bone in children and adults, osteosarcoma, is generally controlled successfully by surgery combined radiotherapy and adjuvant chemotherapy when it is localized. Adriamycin, together with methotrexate and cisplatin, form the backbone of chemotherapy, which commonly termed as “MAP.” Although various active compounds have supplemented conventional chemotherapy, the outcomes for patients with osteosarcoma have not increased for decades^24,25^. Apart from the limited response and adaptive resistance, the acquisition of cross-resistance to multiple active agents contributes to hinder the improvement of MAP-based chemotherapies. We observed that ADM-resistant osteosarcoma cells gained the resistance to paclitaxel and cisplatin, suggesting both U2OS/ADM and MG63/ADM cells developed MDR phenotypes. Consistent with the results, we found that rhodamine 123 accumulation of ADM-resistant cells fell significantly in comparison with the corresponding parental cells. It is well known that P-glycoprotein (ABCB1), MRP1 (ABCC1), MRP2 (ABCC2), and breast cancer resistance protein (ABCG2) played pivotal roles in the regulation of MDR. MRP1, belonging to the superfamily of ATP-binding cassette transporters and conferring resistance to anticancer compounds by exporting drugs in cells^26^, was the most increased expression in these protein. Hence, we selected MRP1 to carried out the following exploration.

An increasing number of studies have illustrated that lncRNA was involved in the regulation of various pathogenesis process in cancer, including cell growth, proliferation, apoptosis, motility, and chemoresistance^3,27^. Notably, several studies have found lncRNA contributes to chemoresistance via multiple signaling cascades in osteosarcoma. Our study found the lncARSR upregulated in ADM-resistant cells and conferred MDR by promoting MRP1 and P-gp expression. A previous study supported our findings, which has shown that lncLUCAT1 promoted MDR related genes MDR1 and MRP5 by sponging miR-200c, conferring resistance to methotrexate^28^. Furthermore, Cheng et al. reported that lncROR triggered P-gp expression, lading to resistance to cisplatin^29^. Conversely, upregulated lncRNA FENDRR expression deprived resistance against ADM via suppressing the expression of P-gp and MRP1^4^. The conflict issues urge more details to clarify the complex roles of lncRNA in the regulation of chemoresistance.

We observed the diminishment of lncARSR inhibited tumor growth and conquered ADM resistance in vivo. The evidence validated the findings in vitro. Prior studies have demonstrated that lncARSR activated AKT through diverse mechanisms. Qu L et al. revealed that lncARSR promoted AKT activation indirectly and led to Sunitinib resistance of renal cancer^10^. Li and colleagues, as well as Ying J et al., proved that lncARSR activated AKT via suppressing PTEN directly^13,30^ Furthermore, lncARSR enhanced AKT activation by promoting YAP1/IRS2/AKT signaling pathways^31^. It is well documented that AKT activation was involved in chemoresistance in various cancer, including ovarian and endometrial cancer, breast cancer, non-small cell lung cell, and osteosarcoma^32^. For example, hypoxia-activated AKT led to overexpression of MRP1 in hepatocellular carcinoma^33^. Transgelin2 motivated AKT and enhanced MRP1 via GSK-3β-dependent pathways in breast cancer^34^. Aberrant expression of glucose transporter 1 enhanced phosphorylation of AKT and promoted Survivin expression, inducing chemoresistance of triplicate-negative breast cancer^35^. Smad4 diminished colorectal cancer cells resistance to 5ʹ-fluorouracil by repressing AKT-Survivin cascades^36^. Activation of AKT triggered MMP2 via various signaling pathways, such as EGFR-AKT-MMP2^37^ and AKT-mTOR-MMP2^38^. The evidence provided a rationale for investigating the effects of AKT-targeted therapy on overcoming the chemoresistance of osteosarcoma. Moreover, the selective PI3K inhibitor BKM120 conquered resistance to chemotherapy by suppression of AKT axis and MDR expression^39^. Many preclinical studies have confirmed the efficiency of PI3K/AKT/mTOR pathways-targeted chemicals on chemo-resistant osteosarcoma; however, only a few mTOR-targeted compounds succeeded in entering clinical trials currently. The conflict between the success of bench studies and the failure of clinical trials indicated the complex network of AKT-modulated chemoresistance remained largely unknown. Our findings that lncARSR conferred ADM resistance and boosted osteosarcoma malignancy through AKT-dependent pathways, at least, fulfilled parts of the gaps between lncARSR and AKT-mediated chemoresistance. Exploration of lncARSR-targeted regimen and AKT inhibitors combination therapy may help improve the clinical outcomes of recurrent or refractory osteosarcoma.

More details are needed to clarify the connection between lncARSR and the clinical-pathological features. First, the distribution of lncARSR in stage-specific osteosarcoma or recurrent/metastatic osteosarcoma remains unknown. Second, the relationship between lncARSR and oncogenes in AKT-related cascades, such as *RAS*, *RAF*, or *SRC*, is a lack of investigation. Third, patient-derived xenograft models are necessary for further evaluating the potential of combination regimens.

Briefly, the current study demonstrated the lncARSR increased in ADM-resistant osteosarcoma cells, induced overexpression of MRP1, Survivin, and MMP2 via activating AKT. Deregulation of lncARSR recovered the sensitivity to ADM. Furthermore, combined sh-lncARSR and ADM magnified the inhibition of tumor growth. Our study extended the knowledge about the involvement of lncARSR in chemoresistance and provided a novel therapeutic target for treating refractory osteosarcoma.
