# Correction: Long noncoding RNA lncARSR confers resistance to Adriamycin and promotes osteosarcoma progression

**DOI:** 10.1038/s41419-021-03779-5

**Published:** 2021-05-07

**Authors:** Peng Shen, Yanfeng Cheng

**Affiliations:** 1 Department of Orthopedics, Shengjing Hospital, China Medical University, 36 Sanhao Street, Heping District, Shenyang, Liaoning 110004 China; 2 Department of Dermatology, Shengjing Hospital of China Medical University, 36 Sanhao Street, Heping District, Shenyang, Liaoning 110004 China

**Keywords:** Bone cancer, Translational research

Correction to: *Cell Death and Disease*

10.1038/s41419-020-2573-2 published online 13 May 2020

Since the publication of the article the authors noticed there was an error in the affiliations for Peng Shen. The correct affiliations for this author are:

^1^Department of Orthopedics, Shengjing Hospital, China Medical University, 36 Sanhao Street, Heping District, Shenyang, Liaoning 110004, China

^2^Department of Dermatology, Shengjing Hospital of China Medical University, 36 Sanhao Street, Heping District, Shenyang, Liaoning 110004, China

